# Mutation of *PUB21* in tomato leads to reduced susceptibility to necrotrophic fungi

**DOI:** 10.1186/s12870-025-07107-3

**Published:** 2025-08-08

**Authors:** Miguel Ramírez Gaona, Ageeth van Tuinen, Danny Schipper, Ángeles Ramos Peregrina, Richard G.F. Visser, Jan A.L. van Kan, Yuling Bai, Anne-Marie A. Wolters

**Affiliations:** 1 Plant Breeding, Wageningen University & Research, Droevendaalsesteeg 1, 6708PB, Wageningen, The Netherlands; 2 Laboratory of Phytopathology, Wageningen University & Research, Droevendaalsesteeg 1, 6708PB Wageningen, The Netherlands

**Keywords:** Tomato, EMS, *PUB21*, Susceptibility gene (S-gene), *Botrytis cinerea*, *Alternaria Solani*

## Abstract

**Background:**

Cultivated tomato is susceptible to necrotrophic pathogens *Botrytis cinerea* and *Alternaria solani*. No dominant resistance against these pathogens has been reported in wild relatives of tomato.

**Results:**

Through screening of a tomato Micro-Tom EMS population we identified a mutant that showed decreased susceptibility to both necrotrophic fungi. Previously, we reported a mutation in the tomato *PUB17* gene as the cause of reduced susceptibility in this mutant. Surprisingly, M4 progeny of one M3 plant homozygous for the *pub17* mutation showed segregation with some plants displaying an even higher level of resistance than the *pub17* mutant. This highly resistant progeny was shown to contain a mutation in tomato *PUB21* in addition to the mutation in *PUB17*. The role of *PUB21* as a susceptibility factor for both necrotrophic fungi was confirmed in RNAi-silenced and CRISPR-mutated transformants.

**Conclusions:**

In this study we identified a new *PUB* gene, *SlPUB21*, involved in susceptibility of tomato to necrotrophic pathogens. We showed that mutation of this gene resulted in increased resistance against these pathogens.

**Supplementary Information:**

The online version contains supplementary material available at 10.1186/s12870-025-07107-3.

## Background

*Botrytis cinerea* is a necrotrophic fungal pathogen that poses a significant threat to a wide range of more than 1400 plant species, including tomato [20]. This pathogen can cause severe damage to crops both pre- and post-harvest, leading to significant economic losses [15, 21, 53]. The success of *B. cinerea* as a pathogen can be attributed to its broad range of virulence factors, high reproductive capacity, and the lack of dominant resistance genes in its hosts [5, 34, 53]. Thus, finding a way to mitigate the infection caused by *B. cinerea* is a pressing issue.

Efforts have been made to understand the infection process of *B. cinerea* on plant hosts to identify key points of vulnerability. The fungus infects the host plant by secreting a multitude of cell death inducing proteins (CDIPs), suppressing the host autophagic response and manipulating the balance between autophagy and apoptosis [5, 34, 50]. This allows the fungus to grow and reach a critical biomass, before releasing compounds that trigger apoptotic cell death and enabling the spread of the infection.

Since tomato as a crop is susceptible to infection by *B. cinerea*, tomato breeders have attempted to identify sources of resistance. Thus far, no gene providing dominant resistance has been identified in tomato. Hence, sources used by breeders for partial resistance against *B. cinerea* have relied on quantitative trait loci (QTLs) found in wild relatives of tomato such as *Solanum chilense*, *S. peruvianum*, *S. chmielewskii*, *S. pimpinellifolium*, *S. habrochaites* and *S. neorickii* [9, 19, 39, 28]. However, none of these QTLs individually or combined could provide the same level of resistance as in the wild relatives [14, 24].

An alternative approach to developing resistance against *B. cinerea*, and potentially other necrotrophic pathogens such as *Alternaria solani*, is focused on disabling susceptibility (S) genes. An S-gene refers to any gene that plays a role in facilitating infection and maintaining the pathogen-host compatibility. [46] classified S-genes into three categories based on the distinct stage of the plant-pathogen interaction they are involved in: host recognition and entry, immune system suppression, and compatibility maintenance to support pathogen proliferation. Since plant S-genes provide advantages to the pathogen, deactivating or reducing the expression of these genes should result in increased plant resistance. Indeed, accumulating evidence demonstrates that a loss-of-function mutation of S-genes in the host may result in resistance against different pathogens [31, 45, 47], making S-genes compelling targets in the search for resistance against necrotrophic pathogens such as *B. cinerea*. In Arabidopsis more than 60 genes involved in the progression of *B. cinerea* infection have been identified (a.o. [6, 11, 12, 31, 32, 40, 46, 51]. However, many S-genes have crucial functions in plant physiological processes, and therefore, a loss-of-function mutation could potentially result in a decrease of fitness of the plant [27].

To identify novel S-genes that facilitate the infection by necrotrophic fungi, we adopted a forward genetics approach by screening a tomato EMS population for resistance against the necrotrophic pathogen *B. cinerea*. Previously, we described an EMS mutant (named M2042) that carries a knock-out mutation in the U-box E3 ubiquitin ligase *PUB17* gene [41]. The loss of function of the *PUB17* gene led to reduced susceptibility towards *B. cinerea* as well as the necrotrophic fungus *A. solani*. However, some of the tomato mutant plants manifested slight autonecrosis depending on genetic background. However, crossing of the *PUB17* mutant with different genetic backgrounds successfully abolished the spontaneous autonecrosis in some of the progeny [41], thus increasing the potential of its use in breeding.

In this study we report the identification of an additional mutated S-gene in one M3 progeny of the M2042 family that conferred resistance to *B. cinerea* and *A. solani*. The responsible gene mutation was identified and verification that the gene functions as an S-gene was obtained through the analysis of RNAi and CRISPR transformants.

## Results

### An EMS-induced mutation in ***PUB21*** (Solyc11g006030) is correlated with reduced susceptibility to ***Botrytis***

**Fig. 1:**
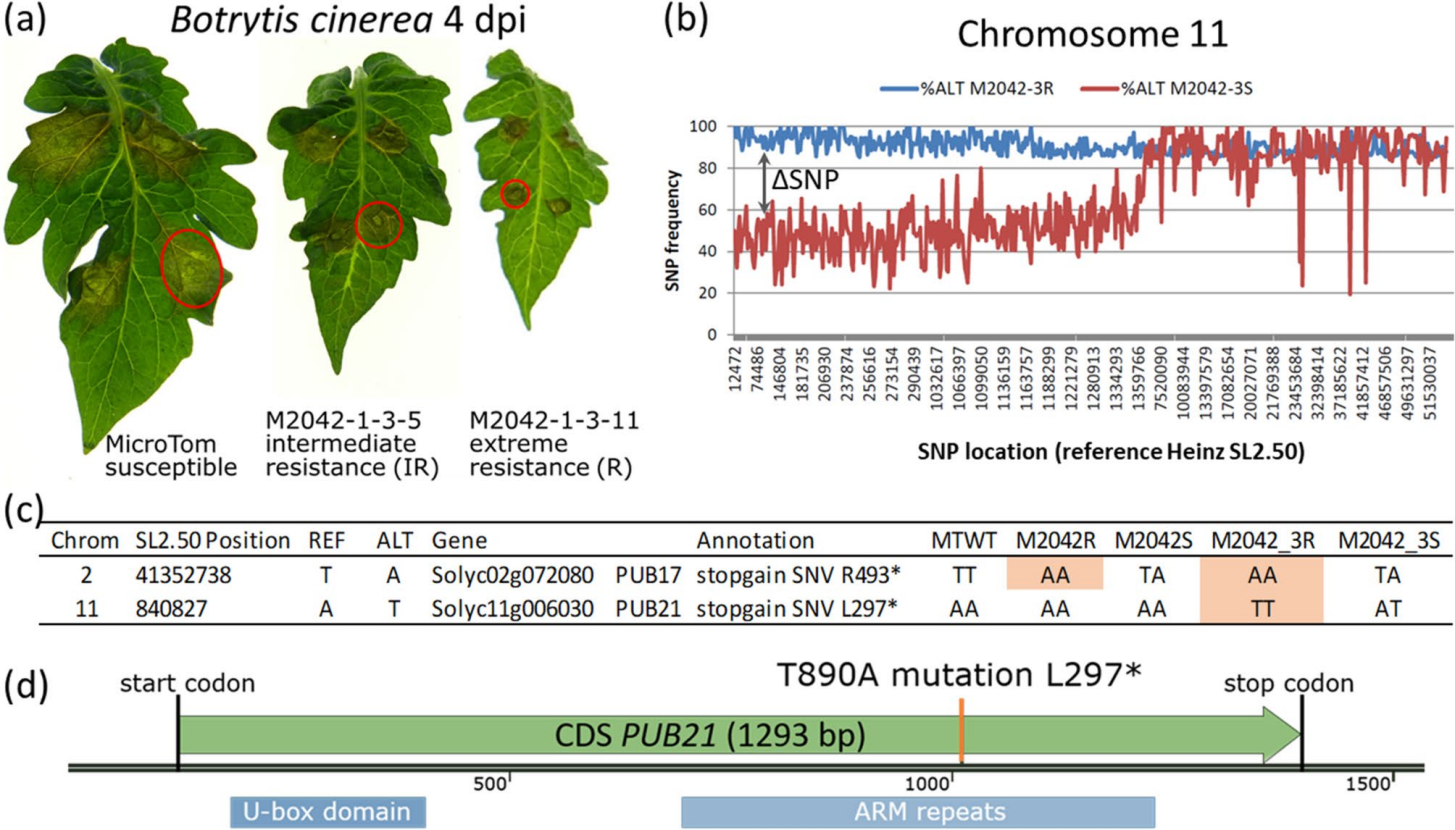
Mutant M2042 candidate gene *PUB21* for reduced susceptibility to *Botrytis cinerea*. (**a**) Three classes of response observed at 4 days after *B. cinerea* inoculation (4 dpi) in wild-type Micro-Tom and M2042-derived M4 plants. (**b**) SNP frequencies of the alternative alleles (compared to reference genome Heinz) in the extreme resistant and the susceptible F2 bulks of mutant M2042-1-3-10 on chromosome 11. (**c**) Chromosome 2 and Chromosome 11 SNP positions per bulk. REF, reference allele from Heinz; ALT, alternative allele; MTWT, wild type Micro-Tom bulk; M2042R, intermediate resistant bulk from M2042-1-2-12; M2042S, susceptible bulk from M2042-1-2-12 [41]; M2042_3R, extreme resistant bulk from M2042-1-3-10; M2042_3S, susceptible bulk from M2042-1-3-10. (**d**) Tomato *PUB21* (Solyc11g006030) has 2 domains: U-box domain (amino acids 22–94) and ARM (armadillo) repeats (amino acids 192–364). The EMS mutation in the ARM repeats domain, indicated with an orange line, causes a premature stop codon L297*

A Micro-Tom EMS population [55] was previously screened for reduced susceptibility to *B. cinerea*. Mutant M2042 displayed intermediate resistance (IR) towards the fungus, and it was shown that the responsible mutation had occurred in gene Solyc02g072080 encoding *PUB17* [41]. Remarkably, when testing progeny of M3 plant M2042-1-3, which was homozygous for the *pub17* mutation (pedigree in Fig. S1), we observed segregation of extreme resistant (R) plants among the M4 progeny. The extreme resistant plants not only displayed smaller lesions but also developed small light green leaves (Fig. 1a) (a.o. M2042-1-3-10, M2042-1-3-11 and M2042-1-3-14) when compared to the IR plants (M2042-1-3-5).This suggested the involvement of another mutation in addition to the one in *PUB17*.

To identify the additional mutation, segregating F2 populations were produced from a cross between MM and M4 plant M2042-1-3-10 (pedigree in Fig. S1 b). Five F1 plants were selfed to produce F2 seeds. A total of 205 F2 plants were screened using a *B. cinerea* detached leaf assay (DLA) to distinguish between three different classes: extreme resistant (small lesion = R), intermediate resistant (medium lesion = IR) and susceptible (large lesion = S) plants. Subsequently, plants showing either very small or very large lesion diameters were selected and reinoculated to confirm resistance or susceptibility. The three groups (S, IR, R) showed a ratio of 9:6:1 suggesting the involvement of two distinct, genetically unlinked loci. Next, two extreme pools were constructed for a bulked segregant analysis (BSA): extreme resistant (M2042-3R) and susceptible (M2042-3 S), with 13 and 14 plants per pool, respectively. DNA of the selected plants was mixed per pool and subjected to Whole Genome Sequencing (WGS). A total of 2,028,009 SNPs were obtained (compared to the reference genome of tomato cv. Heinz) and filtered to identify causal candidate genes. Bioinformatic analyses were performed to select sequence variants absent from wild-type Micro-Tom plants, present in high frequency in M2042-3R and in low frequency in M2042-3 S pool (Fig. 1b). In pool M2042-3R, the stop-gain mutation on chromosome 2 previously identified in *PUB17* was present [41] Fig. 1c), along with an additional mutation on the top of chromosome 11 (Fig. 1b). A mutation consisting of a T→A transversion was observed in gene Solyc11g006030 (Fig. 1c) at position 890 of the coding region, resulting in a premature stop codon L297* (Fig. 1d). Individual plants per pool were genotyped to confirm the correlation between the mutation and *Botrytis* resistance. The Solyc11g006030 gene product belongs to the Plant U-box (PUB) protein family and has similarity with Arabidopsis At5g37490 (PUB21/CMPG5) and potato StPUB21 (Fig. S2). It is therefore proposed as the tomato homolog of At*PUB21*.

**Fig. 2:**
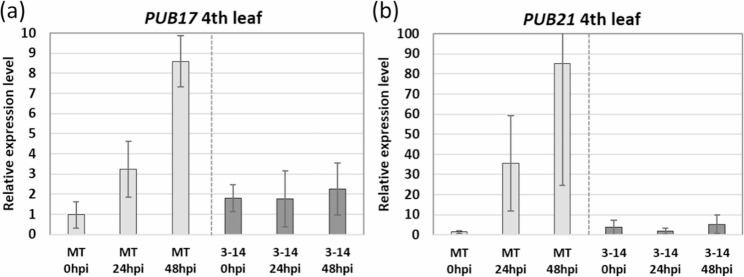
Relative gene expression level of *PUB17* (**a**) and *PUB21* (**b**) in wild-type Micro-Tom (MT) and *Botrytis*-resistant double mutant plant M2042-1-3-14 (3–14) upon infection with *B. cinerea*. hpi, hours post inoculation

To determine the relative expression levels of both genes *PUB17* and *PUB21*, a RT-qPCR was performed using wild-type (WT) MT plants and M4 progeny showing strong resistance (double mutant M2042-1-3-14). Samples of mock- and *B. cinerea-*inoculated leaves were collected at three time points, 0, 24 and 48 h post inoculation (hpi). The expression of both *PUB17* and *PUB21* was highly induced upon infection with *B. cinerea* in WT MT (Fig. 2). The large variation observed in *PUB21* expression in MT at 24 and 48 hpi can be attributed to the lower absolute expression level of *PUB21* reported for WT Heinz leaves under non-inoculated conditions (0.15 RPKM) as compared to *PUB17* gene expression (19.71 RPKM), as shown in the Tomato Expression Database (TED; [23]. Contrasting with WT MT, in the double *pub17*/*pub21* mutant M2042-1-3-14 both *PUB17* and *PUB21* expression remained uninduced after inoculation with *B. cinerea* (Fig. 2).

### ***Botrytis*** resistance in single ***pub17***, single ***pub21*** and double ***pub17/pub21*** mutants

**Fig. 3:**
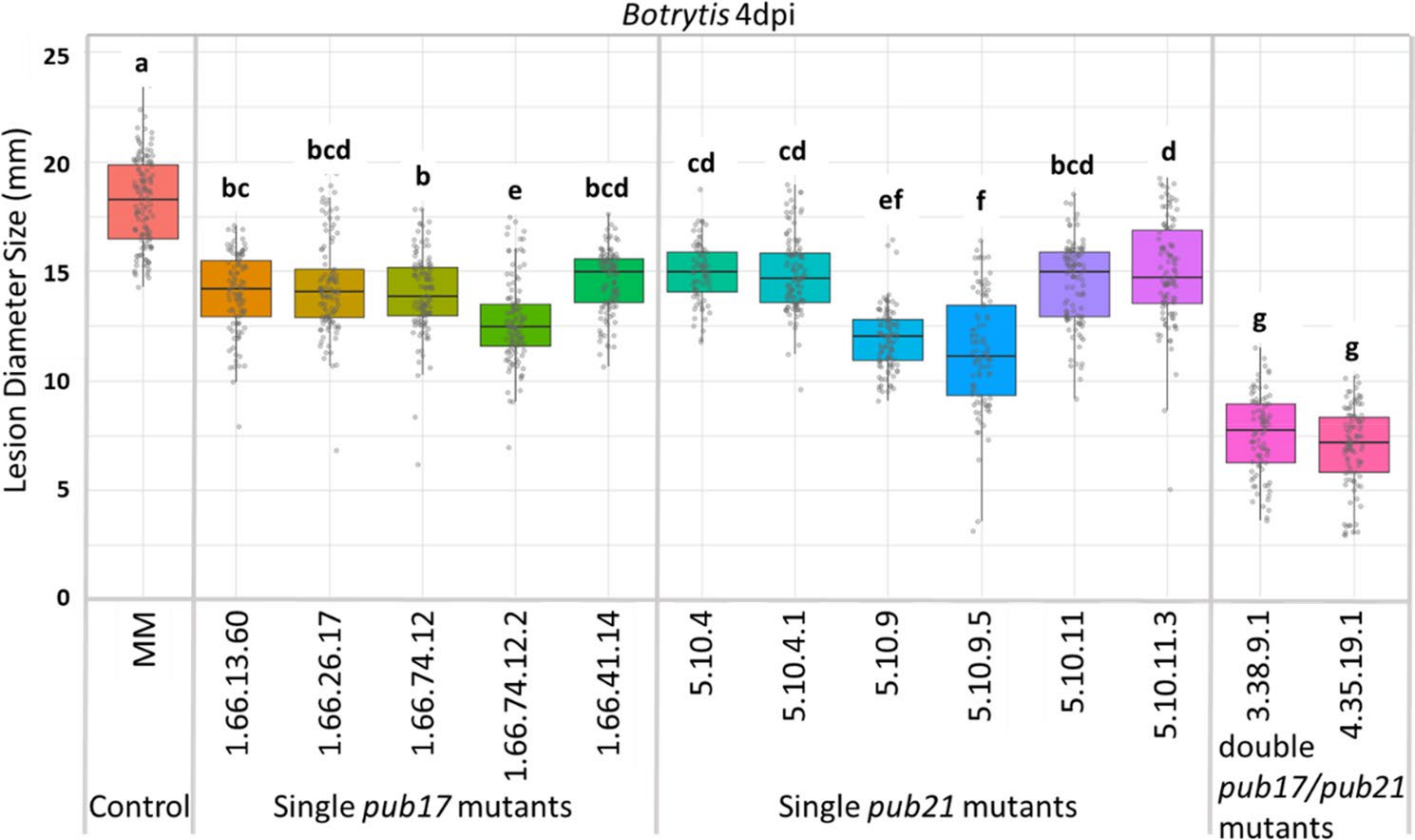
Average lesion diameters of single *pub17* and *pub21* mutants and double *pub17*/*pub21* mutant F4 and F5 families compared with Moneymaker (MM) after *Botrytis* infection. Results from 4 days post inoculation (dpi). Pedigree information of the mutant lines is shown in Fig. S1

To determine the individual effect of the mutation in *PUB21*, F2 plants from the cross between MM and EMS double mutant M2042-1-3-10 were genotyped for MT mutations *d* (dwarf) and *sp* (self-pruning) (Martí et al., [37]) with a PCR using primers shown in Table S1. Selected individuals were self-fertilized to produce progeny with homozygous MM alleles for *D* and *Sp*, in order to obtain progeny with a plant morphology more similar to MM than the dwarf tomato MT. Furthermore, they were selected for being homozygous double *pub17*/*pub21* mutants or homozygous single *pub21* mutants with homozygous MM allele of *PUB17* (Fig. S1 b). Subsequently, three F3 and three F4 single *pub21* lines along with two F4 double mutant lines were screened for *Botrytis* resistance and compared with four F4 and one F5 single *pub17* mutant lines (Fig. S1a) and control MM plants using the DLA approach. Average lesion diameters per line were calculated (Fig. 3).

Overall, the single *pub17* and *pub21* mutants showed comparable average lesion diameters, differing significantly from control MM plants. The single mutants both displayed intermediate *Botrytis* resistance. The double *pub17*/*pub21* mutants showed stronger reduction in average lesion diameters, significantly different from the single mutants, indicating strong *Botrytis* resistance.

### Silencing of ***PUB21*** by RNAi results in increased resistance against ***B. cinerea***

**Fig. 4:**
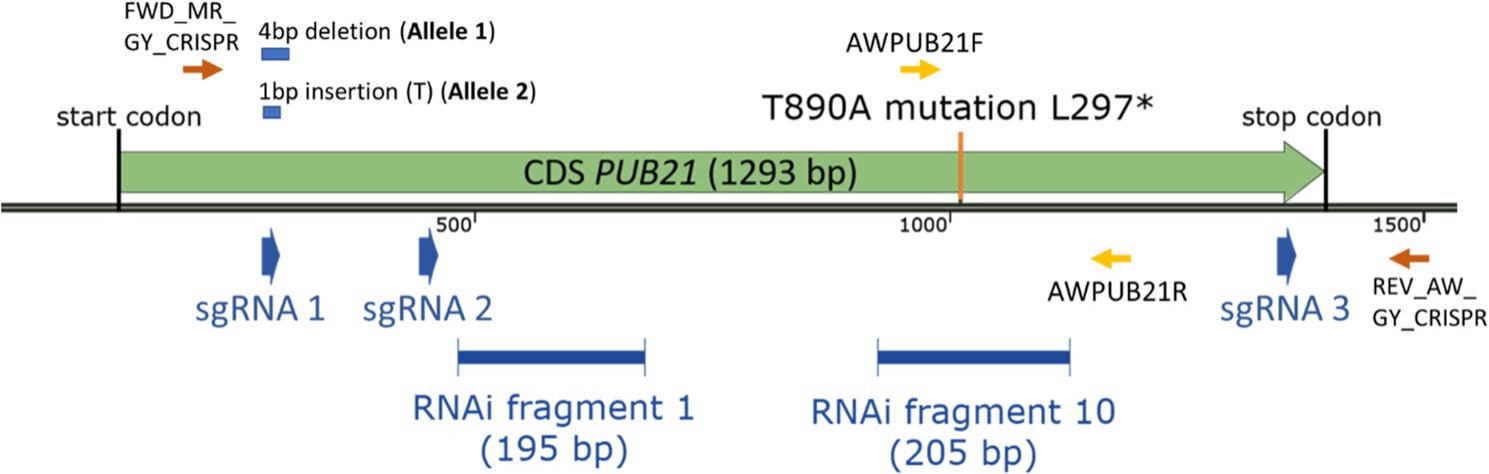
RNAi fragments and CRISPR guide RNA target sites in gene *SlPUB21* (Solyc11g006030). The single exon of the wild-type *PUB21* allele in cultivar Heinz is shown as a green arrow, with underneath the positions of two RNAi fragments for silencing, and three guide RNAs for CRISPR editing as blue arrows. Primers used for analysis of the EMS-induced mutation are shown as small yellow arrows, while the primers used to check for CRISPR-induced mutations are shown as small orange arrows. Sizes of deletions in the CRISPR transformants at the sgRNA1 target site are indicated as small blue boxes above the exon

To confirm that the *PUB21* gene is indeed an S-gene towards *B. cinerea*, knock-down and knock-out transformants in tomato MM background were produced using RNAi and CRISPR editing, respectively. Two RNAi constructs targeting *PUB21* were made: RNAi fragment 1 (195 bp) targeting the region between the U-box domain and ARM repeats, and RNAi fragment 10 (205 bp) targeting the ARM repeats domain (Fig. 4).

**Fig. 5:**
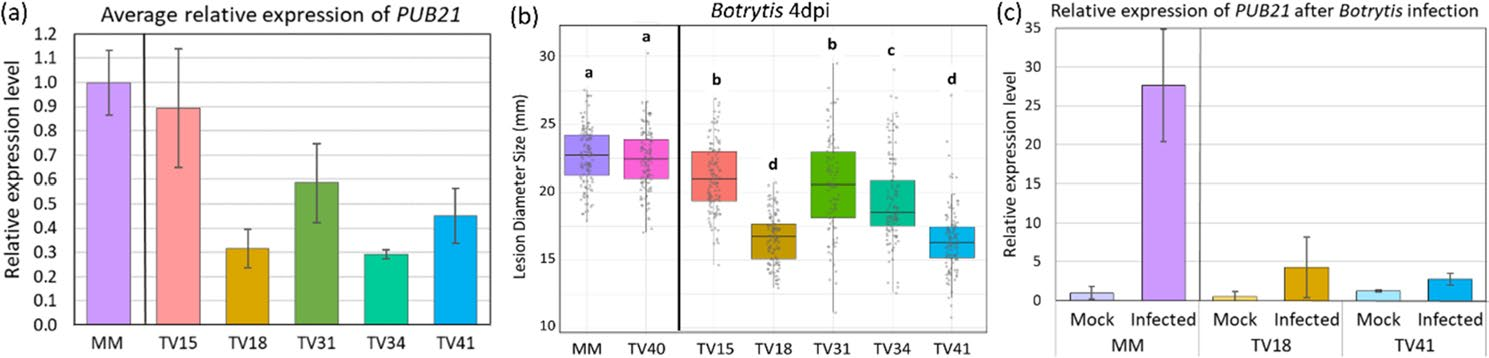
(**a**) Relative expression levels of tomato *PUB21* in RNAi T3 families (TV15-TV41) compared to untransformed Moneymaker (MM) as determined by qRT-PCR using *EF1α* as reference gene. (**b**) Boxplot of *Botrytis cinerea* lesion diameters on leaves from *PUB21* RNAi T3 families with the two controls (MM and TV202240) on the left, results from 4 dpi. (**c**) Relative expression levels of *PUB21* in RNAi T3 families TV18 and TV41 compared to untransformed MM at 45 h post inoculation with *B. cinerea* or mock treatment. Codes of the T3 families are explained in Table 1

A total of 18 RNAi transformants was obtained with construct RNAi1 and 17 with construct RNAi10. After transfer of transformants to the greenhouse it was noticed that some of the RNAi transformants showed slight autonecrosis on the leaves. T2 progeny was obtained from these primary transformants and individual plants were selected for T3 seed production based on the presence of NPTII from the silencing construct. Relative expression levels of *PUB21* were determined in the silenced lines using qRT-PCR (Table 1; Fig. 5a). As mentioned above, absolute expression level of *PUB21* is low in non-infected wild-type plants, which complicated accurate measurement of silencing levels. Despite this, several RNAi transformant T3 families showing a lower level of expression than control plants were identified.

**Table 1:** RNAi *PUB21* transformants and codes of T2 and T3 progeny

RNAi T1 transformant*	T2 plant	T3 Family	Code	Relative PUB21 expression level
RNAi 1–5	TV191052-4	TV202215	TV15	0.89
RNAi 1–7	TV191053-5	TV202218	TV18	0.31
RNAi 1–21	TV191059-20	TV202231	TV31	0.58
RNAi 10 − 9	TV191072-16	TV202234	TV34	0.29
RNAi 10–26	TV191077-11	TV202241	TV41	0.45
RNAi 10–26	TV191077-7	TV202240	TV40 (no NPTII)	ND
Moneymaker control			WT	1.0

* RNAi 1 transformants were obtained with a silencing construct containing RNAi fragment 1 (Fig. 4) while RNAi 10 transformants were obtained with RNAi fragment 10. ND, not determined

The T3 families were inoculated with *B. cinerea* in a DLA. The two controls, MM and TV202240 (no NPTII present), showed similar *B. cinerea* lesion diameters (Fig. 5b). In contrast, significantly smaller *B. cinerea* lesions were observed on leaves from T3 families TV202218, TV202234 and TV202241. In an independent experiment, the T3 families TV202218 and TV202241 showing the highest resistance, together with MM as a control, were inoculated with *B. cinerea* to study the gene expression of *PUB21* after infection (Fig. 5c). At 45 hpi, wild-type MM showed a strong upregulation of *PUB21* gene expression compared to mock treatment, similar to the results for Micro-Tom (Fig. 2). However, the *PUB21* expression levels remained low or non-significantly different after *Botrytis* infection compared with mock treatment in both RNAi families (Fig. 5c).

To generate CRISPR/Cas9 mutations in *PUB21*, a transformation construct was designed with 3 sgRNAs targeting different regions of the gene (Fig. 4). MM was transformed with this construct and 37 transformants were obtained. The transformants were genotyped by sequencing PCR products obtained using a primer pair flanking the first and last sgRNAs, leading to the identification of 2 transformants with mutant alleles at the target site of sgRNA1. No mutations at the target sites of sgRNA2 or sgRNA3 were observed. Although care was taken to select the best 3 sgRNAs for tomato *PUB21* (as described in Materials and methods), the lack of detectable editing at the downstream sites may reflect lower sgRNA efficiency. Of the three sgRNAs, sgRNA1 had the highest score according to sgRNA Scorer 2.0.

Small indels were observed in plants 30 (allele 1) and 35 (allele 2) (Fig. 4). A summary of the identified mutations is provided in Table 2. The CRISPR plants homozygous for mutant *PUB21* allele 1 or 2 had a height similar to MM, with slightly smaller leaves. Small spontaneous necrotic spots appeared in older leaves of these plants.

**Table 2:** Progeny of *PUB21* CRISPR mutants. WT, wild type; NA, not applicable

T1 plant #	T2 family-plant #	T3 family	Code	Mutant alleles	Mutation
30	TV191084-16	TV202269	TV69	Homozygous mutant allele 1	4-bp deletion
30	TV191085-32	TV202278	TV78	Homozygous WT allele	NA
35	TV191086-6	TV202281	TV81	Heterozygous mutant allele 1	4-bp deletion
35	TV191086-30	TV202285	TV85	Heterozygous mutant allele 2	1-bp insertion (T)
35	TV191086-9	TV202283	TV83	Homozygous WT allele	NA

Homozygous mutant T3 progeny was obtained from T1 transformants 30 and 35 (Table 2). The T3 families were subjected to a *B. cinerea* DLA. MM plants and *PUB21* CRISPR T3 families TV202278 (TV78) and TV202283 (TV83) containing only wild-type *PUB21* alleles, were used as the susceptible controls.

**Fig. 6:**
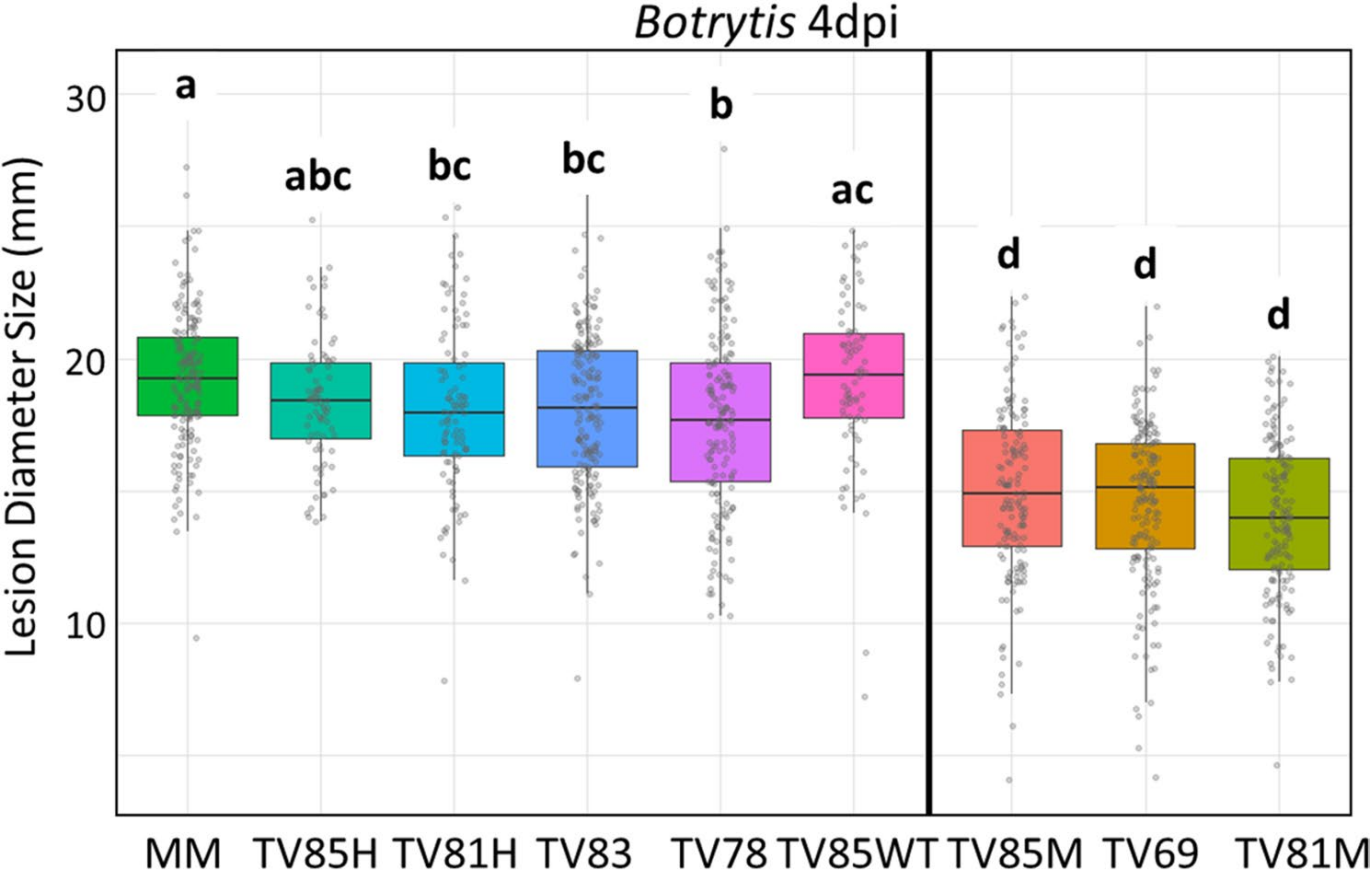
Boxplot of *Botrytis cinerea* lesion diameter sizes on leaves from *PUB21* CRISPR mutant T3 plants of TV85M (homozygous for mutant allele 2), TV69 and TV81M (both homozygous for mutant allele 1), compared with negative controls Moneymaker (MM), TV85H (heterozygous for mutant allele 2), TV81H (heterozygous for mutant allele 1), non-mutant T3 families TV83 and TV78 and T3 plants from TV85WT, 4 days after inoculation. Different letters above the boxplots indicate significant differences, as calculated by Tukey HSD method (*P* < 0.05). H, heterozygous; WT, homozygous wild-type; M, homozygous mutant

Compared with the negative controls (MM and T3 families TV78 and TV83), all T3 plants homozygous for CRISPR-induced mutations in *PUB21* exhibited significantly smaller lesion diameters upon *B. cinerea* inoculation in the DLA (Fig. 6), demonstrating that knocking out *PUB21* in MM background leads to increased resistance to *B. cinerea*. The homozygous family TV69 displayed lesions ~ 24% smaller than the WT MM lesions at 4 dpi. Meanwhile the T3 families TV81 and TV85, segregating for the 4-bp deletion and 1-bp deletion respectively, were split into three groups: homozygous mutants (TV81M, TV85M), heterozygous mutants (TV81H, TV85H) and homozygous wild-type (TV81WT, TV85WT). The homozygous TV81M plants exhibited lesions ~ 27% smaller than the WT MM lesions at 4 dpi, while lesions from TV85M plants were ~ 22% smaller than the WT lesions.

### Consequence of mutations on PUB21 protein structure/architecture

**Fig. 7:**
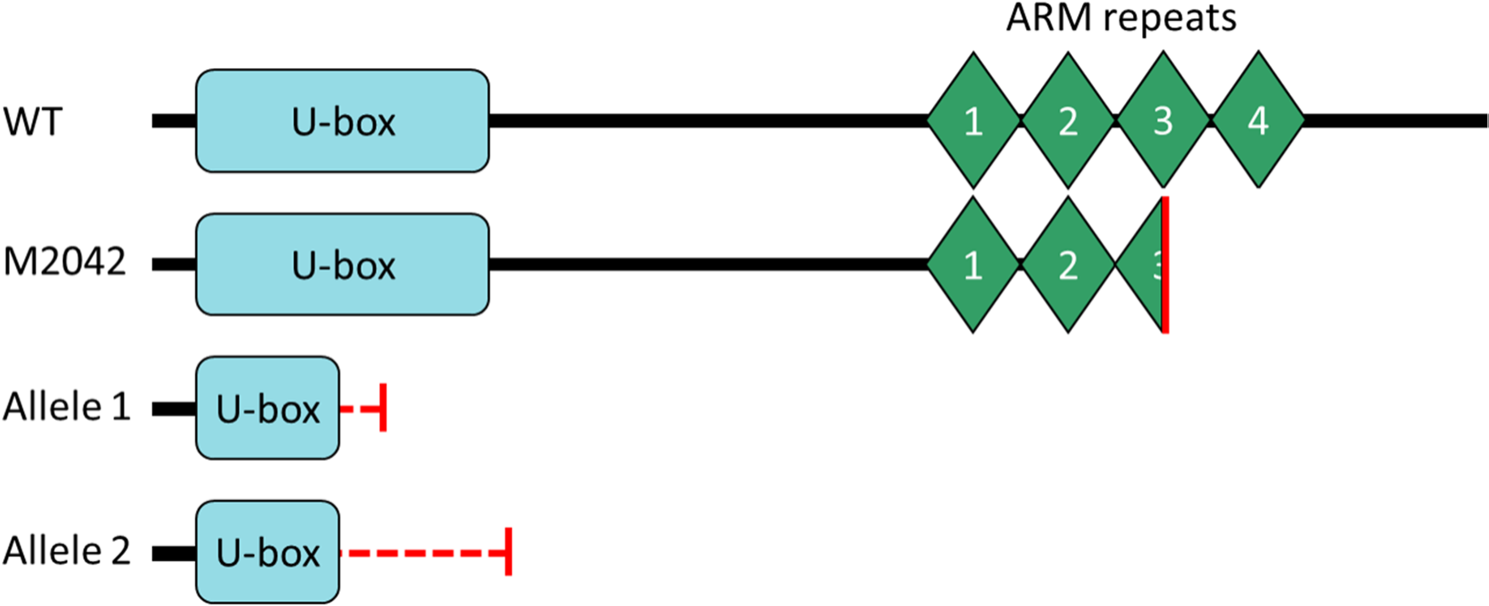
Protein domains in mutant *PUB21* alleles. WT, wild type tomato PUB21 protein; M2042, EMS pub21 mutant protein; Allele 1–2, CRISPR pub21 mutant proteins. Output obtained from the Scan Prosite tool (accessed on 06/06/23). Red vertical lines indicate early stop codons; red dashed horizontal lines indicate different amino acids than in the WT allele

To compare the sequence features of the PUB21 proteins in the stable CRISPR mutant with the original *PUB21* EMS mutant, a multiple sequence alignment was made of the predicted proteins (Fig. S3). The alignment revealed that both CRISPR mutant alleles had frameshift mutations and early stop codons halfway through the U-box domain, leading also to a complete loss of the ARM domain (amino acids 192–364) (Fig. 7). Meanwhile the EMS mutant contained an intact U-box domain (amino acids 22–94) with an early stop codon within the ARM domain, leading to a truncated third ARM repeat and a loss of the fourth ARM repeat.

### Susceptibility of ***PUB21*** mutants to other pathogens

**Fig. 8:**
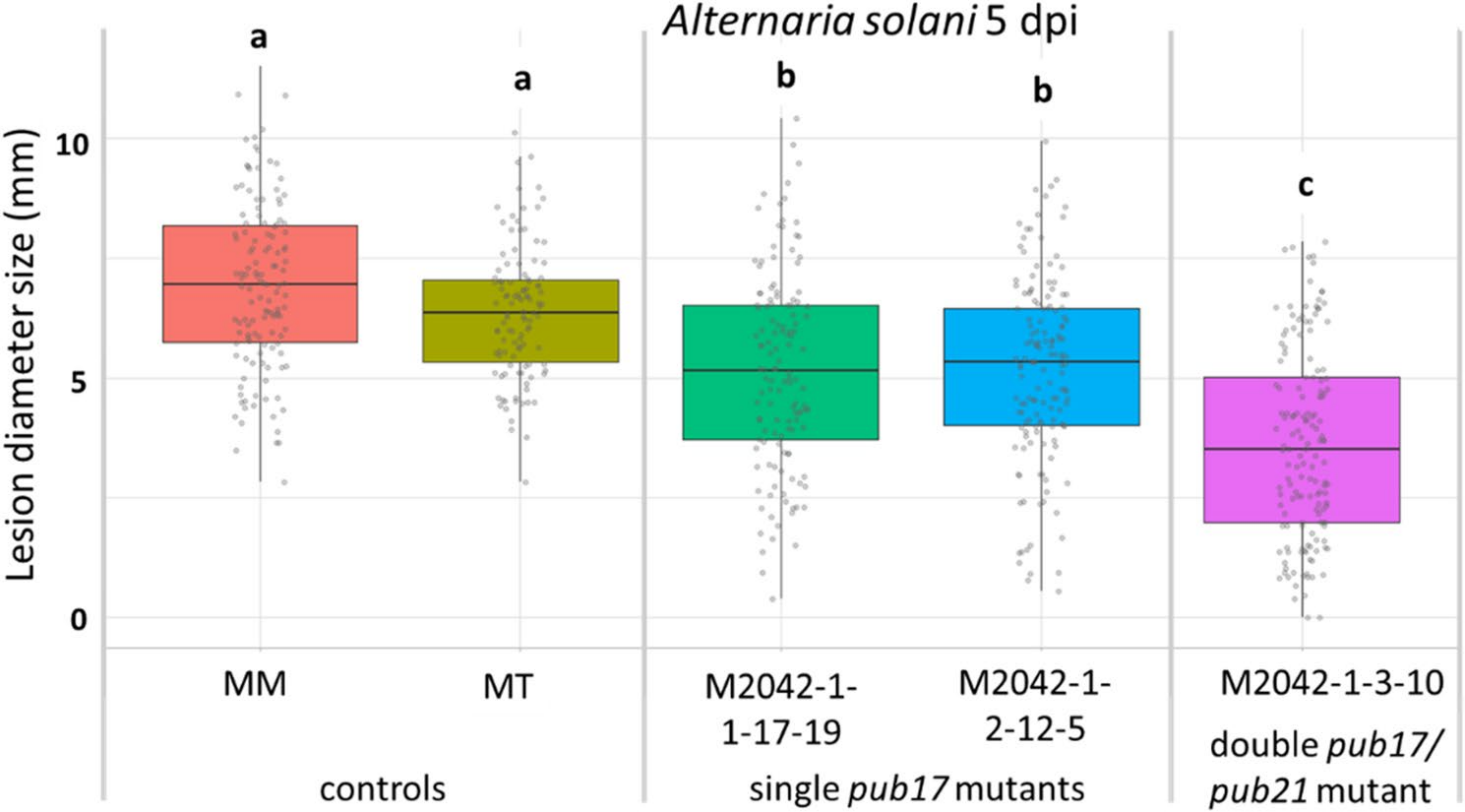
Average lesion diameter sizes on leaves 5 days after inoculation (5 dpi) with *Alternaria solani*. MM, Moneymaker; MT, Micro-Tom. Pedigree of the mutant plants shown in Fig. S1

To investigate whether mutating the *PUB21* gene influences susceptibility to other tomato pathogens, disease assays were performed on the *pub21* mutant with the necrotrophic fungus *A. solani*. The original single *pub17* M2042 mutant [41] as well as the EMS-derived *pub17*/*pub21* double mutant showed significantly reduced lesion diameters (Fig. 8), with the *pub17*/*pub21* double mutant showing smaller lesions than the original M2042 mutant.

**Fig. 9:**
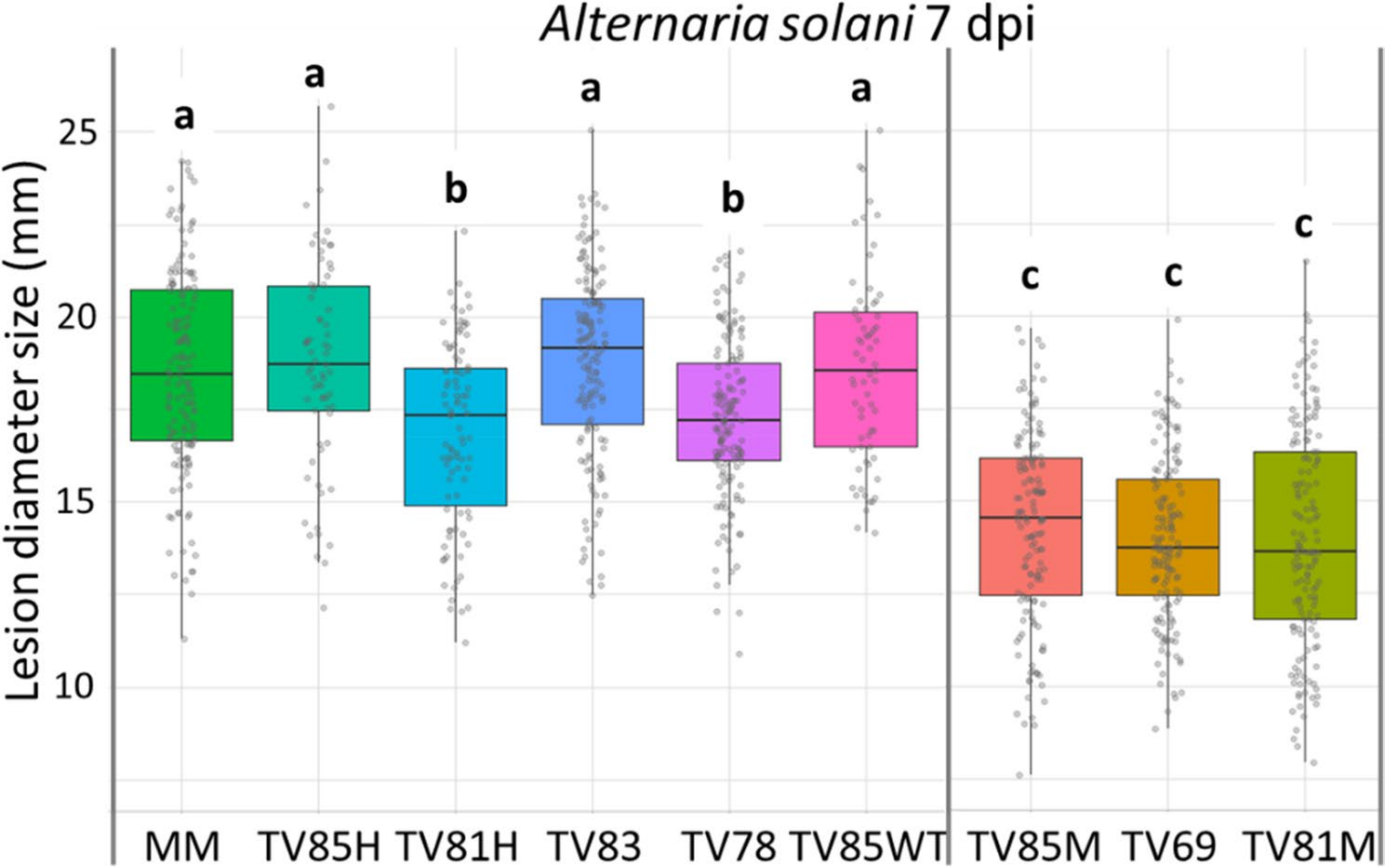
Boxplot of *Alternaria solani* lesion diameters on leaves from *pub21* CRISPR T3 families including controls. Abbreviations of T3 families explained in Table 2. Results from 7 days post inoculation (dpi). Different letters above the boxplots indicate significant differences, as calculated by Tukey HSD method (*P* < 0.05)

In addition, *pub21* CRISPR T3 mutant families and *pub21* T3 RNAi lines were subjected to a DLA with *A. solani*. Homozygous mutant T3 plants of CRISPR families TV202285 (TV85M), TV202269 (TV69) and TV202281 (TV81M) clearly developed significantly smaller lesions than the negative controls (Fig. 9). The RNAi T3 families TV202218, TV202234 and TV202241 displayed significantly smaller lesions compared to the negative controls, MM and T3 family TV202240, while these did not show any significant difference among each other (Fig. S4).

When tested with the obligate biotrophic tomato powdery mildew pathogen *Pseudoidium neolycopersici*, plants of both the original *pub17* mutant M2042 and *pub21* CRISPR T3 family TV202269 were as susceptible as the control plants of MT or MM (Fig. S5).

## Discussion

### Tomato genes ***PUB17*** and ***PUB21*** are susceptibility genes towards necrotrophic fungi

In this study we report that tomato *PUB21*, a U-box E3 ubiquitin ligase [3], acts as a S-gene during infection with the necrotrophic fungi *B. cinerea* and *A. solani*. In Arabidopsis, 64 *PUB* genes have been described [2] and categorized into 7 classes based on the presence/absence of domains other than the highly conserved U-box domain, such as Armadillo repeats (ARM) [48]. PUB21 belongs to Class IV with the presence of ARM repeats located at the C-terminus.

M4 progeny of EMS mutant family M2042-1-3, which showed stronger resistance against *B. cinerea* than the original mutant, contained a mutated *PUB21* allele in addition to the mutation in *PUB17*. The reduced susceptibility observed in the individual F3 and F4 *pub21* mutants was comparable to that observed in the F4 and F5 *pub17*. The role of *PUB21* in conferring susceptibility to *B. cinerea*, independent of *PUB17*, was confirmed through CRISPR mutation and RNAi silencing of the wild-type allele in MM background. Meanwhile, the *pub17/pub21* double mutants showed even lower susceptibility to *B. cinerea.* These results demonstrate that mutations in *PUB17* and *PUB21* have an additive effect on *Botrytis* resistance. In addition, similar to the *pub17* mutants, tomato *pub21* mutants also showed lower susceptibility to the necrotroph *A. solani*. The *pub21* mutation in the EMS mutant resulted in the loss of the two C-terminal Armadillo repeats while the CRISPR-induced mutations resulted in a truncated U-box domain along with the complete loss of the ARM domain. The U-box is a highly conserved domain known mainly for its involvement in pairing with specific E2 proteins providing a certain level of target specificity [48]. Mutations in the U-box domain have been reported to cause a loss of E3 activity [49]. On the other hand, the ARM repeats are involved in protein-protein interactions for plant development, morphogenesis and hormone signaling [10]. Specifically, ARM repeats present in PUB proteins provide a secondary level of specificity when it comes to interacting with E2 ubiquitin-conjugating enzymes, as E2-E3 pairing is determined by both the U-box and the ARM domain [43, 49]. Therefore, loss of these domains would hinder the ubiquitination process as specific binding of proteins targeted by PUB21 is lost. While the CRISPR mutants have a truncated U-box domain and a complete loss of the ARM repeats, two essential domains for proper E3 ligase activity, the reduced susceptibility is similar to that of the EMS mutant. This suggests that the loss of the ARM repeats is enough to lead to lower susceptibility.

Not only truncation of the tomato PUB21 protein results in increased resistance against necrotrophic fungi, but also significantly reduced *PUB21* expression, as shown by the results of the RNAi families. When pathogen-induced expression of *PUB21* in these RNAi plants remains below a certain threshold the infection is less successful than in wild-type plants, confirming the role of PUB21 as susceptibility factor for these necrotrophic pathogens.

### PUB21 and its close homolog PUB20 act as regulators of immunity

Studies on the function of PUB21 in tomato or PUB21 orthologs in other plant species are scarce. Comparison of homologs may provide valuable information on the evolutionary relationship between species and insights on conserved biological pathways. In Arabidopsis, PUB21 is also known as CMPG5. The closest homolog to PUB21 in Arabidopsis is PUB20/CMPG1 (Fig. S2). [56] performed a microarray study in Arabidopsis measuring transcript level changes after pathogen-derived elicitor treatment. *AtPUB21* was induced by treatment with various pathogens, including *B. cinerea*, *Phytophthora infestans*, *Pseudomonas syringae* pv. *phaseolicola*, as well as several PAMP (pathogen-associated molecular pattern) signals [56]. Given the response to pathogens and pathogen-derived molecules, AtPUB21 was suggested to be a regulator of immunity. The different time points of induction in response to different pathogen inoculations suggests that this regulatory function of AtPUB21 depends on the stage of infection. A recent article by [57] showed that both AtPUB20 and AtPUB21 act as negative regulators of immunity at the early stage after *Pseudomonas syringae* pv. *tomato* DC3000 invasion. Both *pub20* and *pub21* mutants showed increased resistance to this bacterial pathogen.

In soybean (*Glycine max*) a *PUB20* gene (*Glyma.14G212200*) and a *PUB21*-like gene (*Glyma.18G042100*; Fig. S2) were among the highest upregulated genes after inoculation with the necrotrophic fungus *Sclerotinia sclerotiorum* [52]. On the other hand, *Nicotiana benthamiana* CMPG1 and the *N. tabacum* ortholog ACRE74 have been reported as a positive regulators of immunity against *Cladosporium fulvum* based on the observation that RNAi-silenced plants exhibited reduced cell death induction triggered after recognition of *C. fulvum* effector Avr9 by resistance gene *Cf-9* [26].

The phylogenetic tree (Fig. S2) shows that the PUB20 and PUB21 proteins from Solanaceous species form two separate clusters, while AtPUB20 and AtPUB21 are in their own subgroup, suggesting that homology between the tomato and Arabidopsis *PUB21* genes is not enough to infer a function from one species to the other. In the case of *A. thaliana*, the amino acid sequences of AtPUB20 and AtPUB21 display 56% identity and 72% similarity. Importantly, they do not interact with the same targets, as AtPUB20 interacts with AGB1 (GTP-binding protein beta 1), while AtPUB21 does not [33]. AGB1 is reported to be required for resistance against the necrotrophic fungus *Plectosphaerella cucumerina* [16]. Evolutionary pressures may have prompted PUB gene E3 ligase diversification to manage different biotic or abiotic stresses.

### Role of tomato PUB21 in programmed cell death

As the loss of function of tomato *PUB21* leads to lower susceptibility to necrotrophic fungi, we can deduce that SlPUB21 functions as a negative regulator of immunity for this class of pathogens. The presence of spontaneous necrotic spots in older leaves of the *pub21* mutants suggest a regulatory function in programmed cell death (PCD). PCD plays a pivotal role in enabling a successful infection of necrotrophic pathogens. Two types of PCD can be distinguished: apoptosis and autophagy. While the activation of either pathway leads to the appearance of necrotic plant tissue, the final outcome of each pathway leads to different effects on plant health. Research on the necrotrophic fungi *Sclerotinia sclerotiorum* and *B. cinerea* has shown that infection benefited from apoptotic PCD triggered upon inoculation with the pathogen [17, 50] proposed that necrotrophic pathogens have the ability to temporarily suppress autophagic cell death in the early phases of infection in order to proliferate and infect the host plant. It remains to be determined which host target genes are involved in PCD regulation [5, 34].

We infer that *PUB21* is one of those host genes. In the *pub21* mutant the balance is shifted towards autophagic PCD. PUB21 may either positively regulate apoptosis or negatively regulate autophagy. We have shown that loss of ARM repeats of PUB21 is sufficient to reduce susceptibility to necrotrophs. Although the specific targets of PUB21 are unknown, studying the protein-protein interactions might reveal the target protein(s) of PUB21 and thereby explain how it contributes to PCD.

### PUB17 and PUB21 are involved in different PCD pathways

An increased resistance against both *Botrytis* and *Alternaria* was observed in the *pub17*/*pub21* EMS double mutant compared to the single *pub17* or *pub21* mutants. Therefore, the double mutant shows an additive effect, suggesting that each gene targets a different part of the complex cell death process. If both genes would be involved in the same pathway we would expect similar lesion diameters when comparing the double mutant with the single mutants. However, the loss of two genes involved in different aspects of the regulation of PCD, leading to stronger resistance against the necrotrophic pathogens, is accompanied by stronger pleiotropic effects on development/growth of the plant. Further testing of the double mutant and the single mutants is thus required to elucidate the roles of both PUB17 and PUB21 in PCD.

## Conclusion

Our results show that *SlPUB21* is a key player in the susceptibility of tomato plants to the necrotrophic pathogens *B. cinerea* and *A. solani*. The mutation of *SlPUB21* provides broad-spectrum resistance to these pathogens without displaying severe pleiotropic effects. The double mutant of *pub17/pub21* in tomato displayed a higher level of resistance, however, it also exhibited reduced plant size. We surmise that SlPUB21 is involved in programmed cell death regulation in a different way than SlPUB17.

## Materials and methods

### Plant material

Two different tomato cultivars were used for this study: cv Micro-Tom (MT) and cv. Moneymaker (MM). MT seeds were obtained from Beekenkamp Plants B.V. (Maasdijk, The Netherlands). MT was chosen to generate an EMS population [55] because of its small size, short life cycle and the possibility to grow at high density [38]. Full details on the development of the MT-EMS population have been described by [41].

### Disease assays

Detached leaf assays (DLA) for both *B. cinerea* strain B05.10 and *A. solani* isolate CBS 143772 were performed following the methods described by [41]. To check the *PUB21* gene expression levels in the *PUB21* RNAi T3 families after *Botrytis* infection, four plants per family or control MM were grown. Per plant opposite side leaflets of one compound leaf were used. One leaflet was used for *Botrytis* inoculation, while the other leaflet was mock-inoculated. For both treatments four droplets were used per leaflet. Samples were obtained 45 h post inoculation by taking 9 mm punches that included the inoculation spots. The powdery mildew disease assay with *Pseudoidium neolycopersici* isolate On-Ne was performed by spraying whole plants with a conidiospore suspension of 2.5 × 10^4^ spores per ml, as described by [4].

### Identification of mutant gene by Bulked Segregant Analysis combined with Whole Genome Sequencing (BSA-WGS)

EMS mutant M2042 [41] showing reduced susceptibility to *B. cinerea* was selfed until M4 plants were obtained (Fig. S1). Individual M4 plants were selfed for M5 seed production (M2042-1-1-17 and M2042-1-2-12) and/or crossed with MM to develop F1 seeds (M2042-1-2-12, M2042-1-3-10). F1 plants were selfed and F2 seeds were collected. F2 plants were tested for susceptibility to *B. cinerea* by DLA to build two pools, consisting of resistant and susceptible F2 plants. Mutants obtained from the F2 generation were selfed to establish F3 lines, and subsequently F4 and F5 lines (Fig. S1a). DNA of the resistant and susceptible F2 plants was isolated following the method described by [41] and subsequently pooled equimolarly, resulting in two DNA pools M2042-3R and M2042-3 S. Two additional pools had been made previously from the F2 progeny of M2042-1-2-12, M2042S (susceptible pool) and M2042R (intermediate resistant pool) [41]; together with a pool of wild-type Micro-Tom plants (MTWT) these were used as controls for the *PUB17* mutation. Whole genome sequencing of the DNA pools was done as outlined in [41]. SNP detection was performed using SAMtools on the reads that were mapped to the tomato Heinz reference genome (version SL2.50).

For each SNP the number of reads containing the reference allele (Heinz) and the number of reads with the alternative allele for each pool were recorded. Subsequent calculations and SNP filtering were performed as described by [41]. Each chromosome was inspected for the presence of large difference of percentage of alternative allele per SNP position between M2042-3R and M2042-3 S (∆SNP = %ALT[R] - %ALT[S] > 50). On the short arm of chromosome 11 (SL2.50ch11:841716..840424), a large ∆SNP was observed between pools M2042-3R and M2042-3 S. The next filtering consisted of selection of SNP positions in exons of annotated genes for which the alternative allele was present in the pools M2042-3R and M2042-3 S of the extreme resistant M2042-1-3-10 but not in the MTWT pool, nor in the previously obtained resistant and susceptible pools (M2042R and M2042S) used to identify *PUB17* [41].

### Gene expression level by RT-qPCR

Gene expression levels were determined by performing a RT-qPCR on plant cDNA synthesized using an iScript cDNA Synthesis kit (BioRad) or TaqMan™ Reverse Transcription Reagents kit (Invitrogen) on RNA extracted through an RNeasy Plant Mini Kit (Qiagen). Specific primers were developed for each gene (Table S1). Elongation factor 1 alpha (*Ef1α*) was used as reference gene. RT-qPCR was performed using a CFX96 Real-Time PCR machine (BioRad) with two technical replicates used per sample. Relative expression of *PUB17* or *PUB21* was calculated with the ΔΔC_T_ method [35].

### RNAi and CRISPR transformation for confirmation of candidate gene

*PUB21* RNAi constructs were generated using the binary vector pHellsgate8 (Helliwell et al. [29]); full details are described in [41]. This vector contains a CaMV 35 S promoter driving the expression of the inverted repeat and a kanamycin resistance gene NPTII as a selectable marker. Primers were designed for *PUB21* to amplify fragments from tomato MM gDNA. Primer sequences are shown in Table S1. RNAi fragment 1 targets the region between the U-box domain and the Armadillo (ARM) repeats of the PUB21 protein, while the RNAi fragment 10 targets the ARM repeats domain.

A CRISPR/Cas9 construct was developed to create deletions within the *PUB21* coding sequence, using 3 sgRNAs alongside the Cas9 endonuclease gene and the NPTII plant selectable marker. The sgRNAs were designed as described by [45]. To select the best 3 sgRNAs, in addition to sgRNA scorer (https://sgrnascorer.cancer.gov [8]), two additional tools were used: GPP sgRNA Designer (https://portals.broadinstitute.org/gpp/public/analysis-tools/sgrna-design [44]), and WU-CRISPR (http://crispr.wustl.edu [54]). The construct was assembled using a Golden Gate cloning system [22] with plasmids from Addgene as described by [45]. The plasmids were cloned using *E.coli* DH5α.

The two RNAi constructs and one CRISPR/Cas9 construct for *PUB21* were transformed into electrocompetent *Agrobacterium tumefaciens* AGL1 + virG cells. Transformation of tomato cv. MM was carried out as previously described [30]. DNA was isolated from young leaves using CTAB buffer (1 M Tris-HCl pH 7.5, 0.5 M EDTA pH 8.0, 5 M NaCl, 2% CTAB) essentially as described by [18]. To confirm the integration of the T-DNAs of the silencing constructs in the genome of the RNAi transformants, a PCR was performed to detect the presence of the *NPTII* gene and 35 S promoter using DreamTaq DNA polymerase (Thermo Scientific, Bleiswijk, The Netherlands). Primer sequences are shown in Table S1. To determine the presence of mutations in the CRISPR transformants PCRs were performed using primers flanking the sgRNA target sites. The PCR products were sent for sequencing to Macrogen Europe (Amsterdam, The Netherlands).

#### Statistical analysis

Data points for each DLA experiment were subjected to an ANOVA F-test using R studio v 1.1.463 [1]. The ANOVA test was followed by a Post Hoc test using Tukey HSD method to perform multiple pairwise-comparisons. Differences were considered significant at *P* < 0.05.

### Protein alignment and prediction tools

Sequencing of the *PUB21* EMS mutant and CRISPR T3 mutants was performed using primers covering the complete genomic sequence (Table S1). MM and MT DNA samples were taken along as controls. The *PUB21* sequences were assembled by aligning the DNA fragments using the plasmid editor ApE [13]. Each complete DNA sequence was translated to RNA using the ExPASy translate tool (https://web.expasy.org/translate/ [25]). Protein alignment was done using the multiple sequences alignment program Clustal Omega provided by EMBL-EBI [36]. Protein domains were predicted using the ScanProsite tool [7]. The Arabidopsis PUB protein sequences were obtained from TAIR (https://www.arabidopsis.org/ [42]), while the rest of the PUB protein sequences were obtained from NCBI (https://www.ncbi.nlm.nih.gov/). After protein alignment, MAFFT7 (https://mafft.cbrc.jp/alignment/server/) was used to build the phylogenetic tree.

## Electronic supplementary material

Below is the link to the electronic supplementary material.

Supplementary Material 1

## Data Availability

The data generated and analyzed supporting the findings of the current work are available within the manuscript and its supplementary information files.
