# Supplementary material for: Mutation of PUB21 in tomato leads to reduced susceptibility to necrotrophic fungi
Source: BMC Plant Biol. 2025 Aug 8;25:1038. doi: 10.1186/s12870-025-07107-3 (PMC12333220; doi:10.1186/s12870-025-07107-3)
Supplement: Supplementary file 1 — Supplementary Material 1 [file 12870_2025_7107_MOESM1_ESM.docx]

**Supplementary material**

**Mutation of *PUB21* in tomato leads to reduced susceptibility to necrotrophic fungi**

Miguel Ramírez Gaona^1^, Ageeth van Tuinen^1^, Danny Schipper^1^, Ángeles Ramos Peregrina^1^, Richard G.F. Visser^1^, Jan A.L. van Kan^2^, Yuling Bai^1^, Anne-Marie A. Wolters^1*^

^1^ Plant Breeding, Wageningen University & Research, Droevendaalsesteeg 1, 6708PB Wageningen, The Netherlands

^2^ Laboratory of Phytopathology, Wageningen University & Research, Droevendaalsesteeg 1, 6708PB Wageningen, The Netherlands

**Supplementary figures**


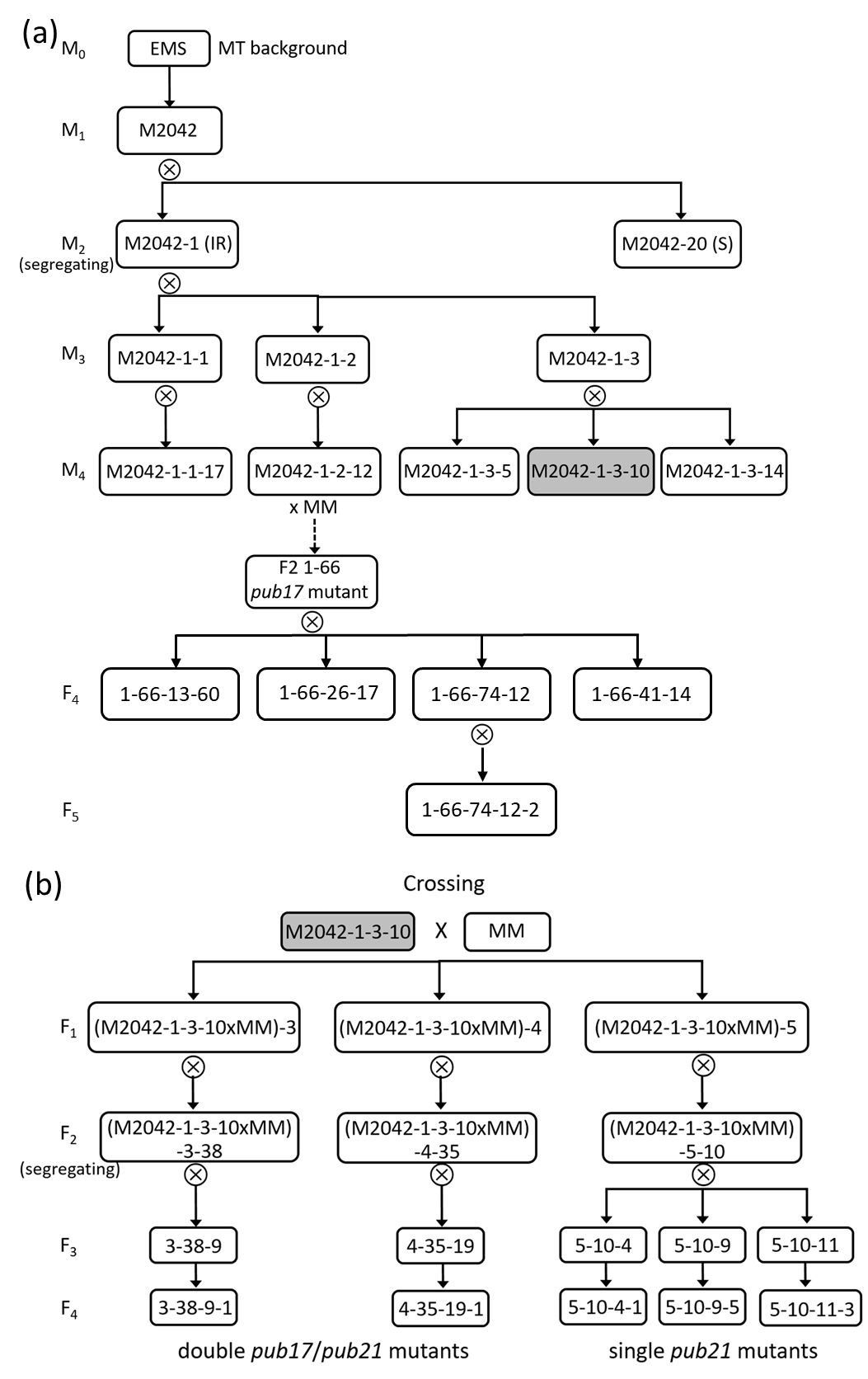


**Fig. S1** Pedigree of M2042 mutant. (**a**) Selfing generations of M2042 in Micro-Tom (MT) background. (**b**) Crossing generations after crossing resistant M4 plant M2042-1-3-10 with Moneymaker (MM). IR, intermediate resistance; S, susceptible.


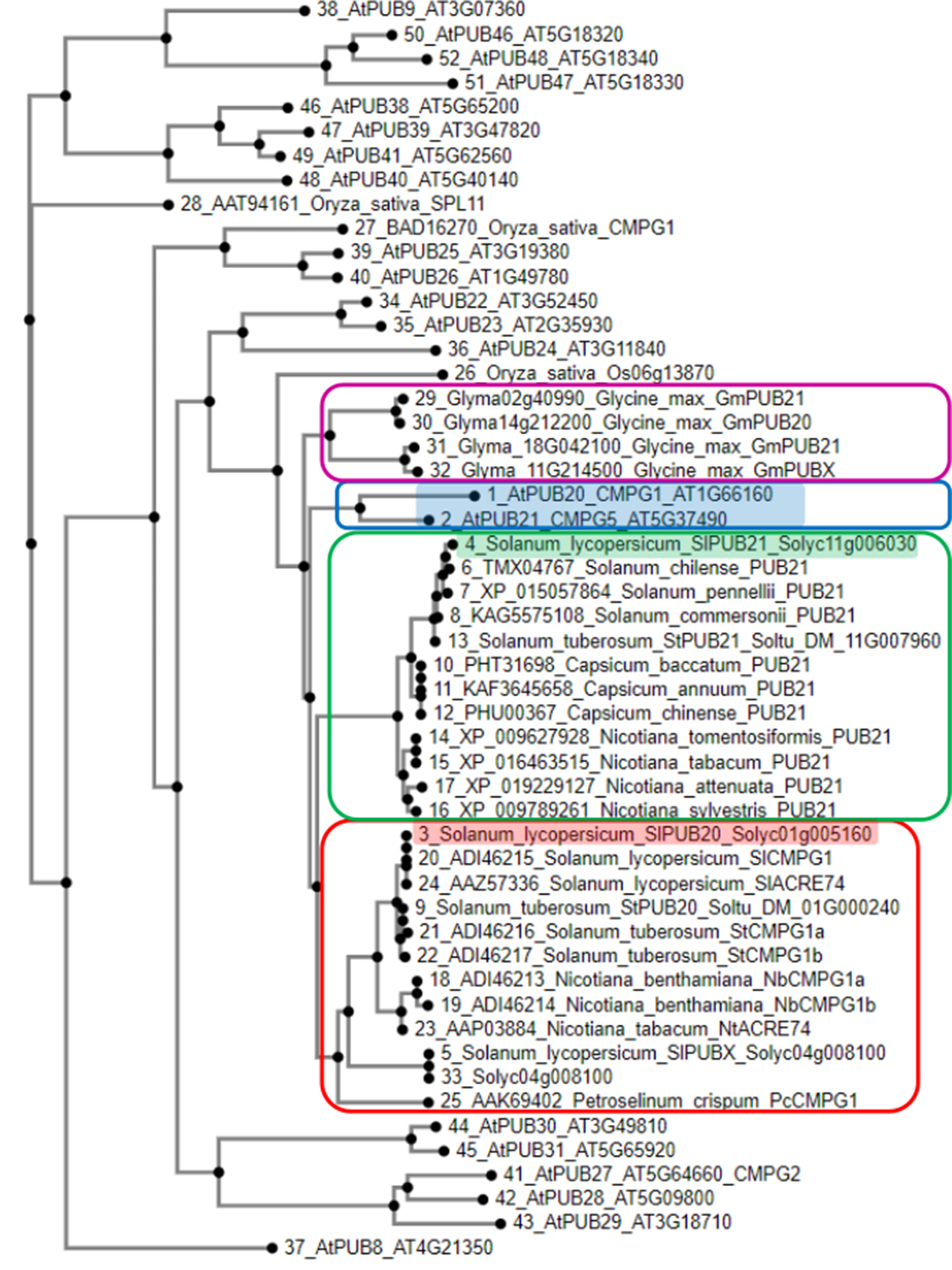


**Fig. S2** Phylogenetic tree of PUB20, PUB21 and Arabidopsis clade IV PUB proteins with short ARM repeats. Arabidopsis PUB20- and PUB21 proteins are shaded in the blue box. The Solanaceous PUB21 proteins are clustered in the red box, while the PUB20 proteins are clustered in the red box. Soybean (*Glycine max*) PUB20/PUB21 orthologous protein are clustered in the purple box. Protein sequence alignment and phylogenetic tree construction were performed using MAFFT7.


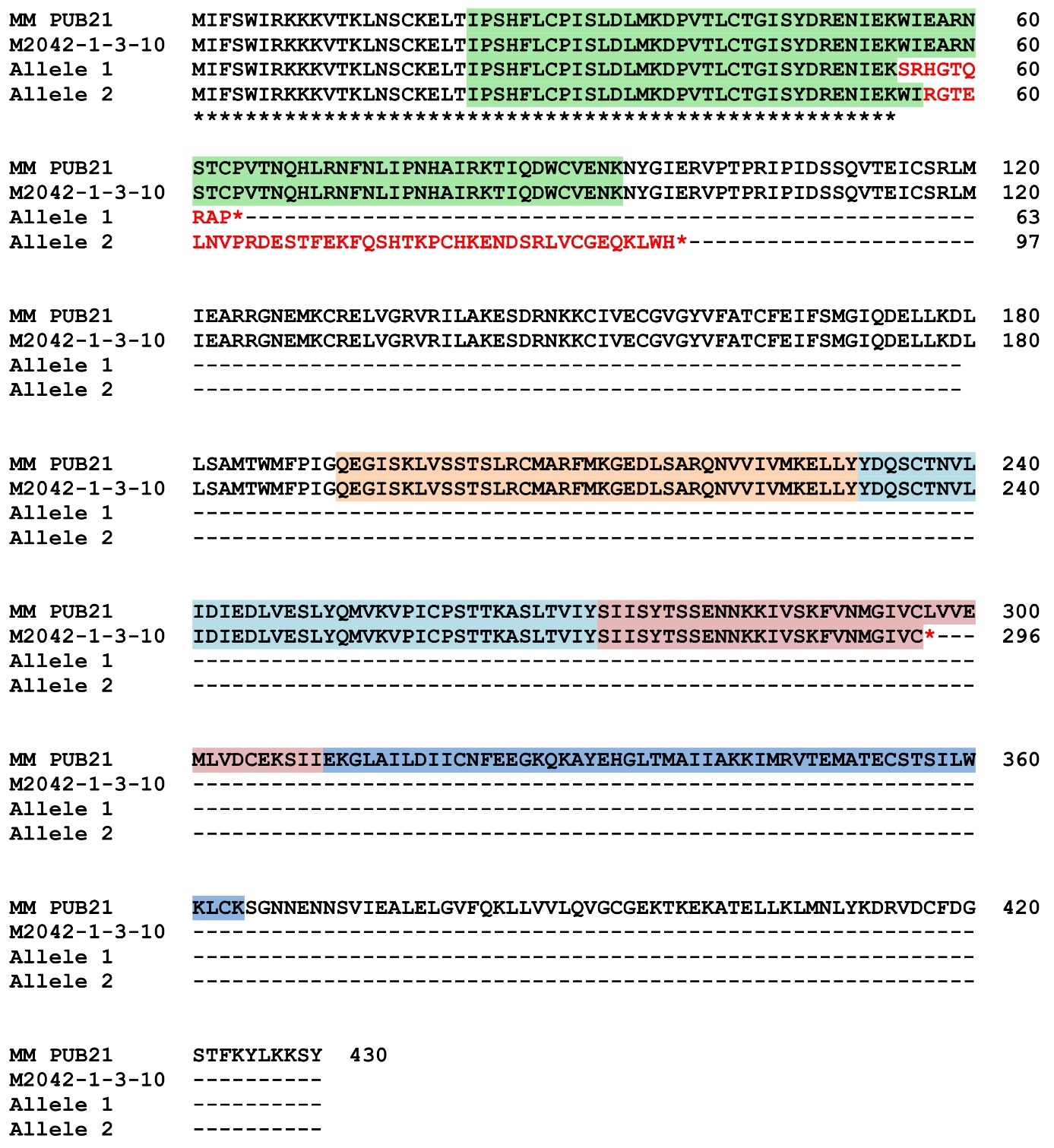


**Fig. S3** Multiple protein sequence alignment of *S. lycopersicum* Moneymaker (MM) wild-type and mutant *PUB21* alleles (EMS mutant M2042-1-3-10, and CRISPR alleles 1-2) using MAFFT7 multiple sequence alignment. Predicted coding sequence from each complete DNA sequence was translated to protein using the ExPASy translate tool (Gasteiger et al. 2003). The U-box domain is highlighted in green while the four ARM domains are highlighted in orange, light blue, pink and dark blue. Amino acids deviating from the wild-type protein are indicated with red letters.

**
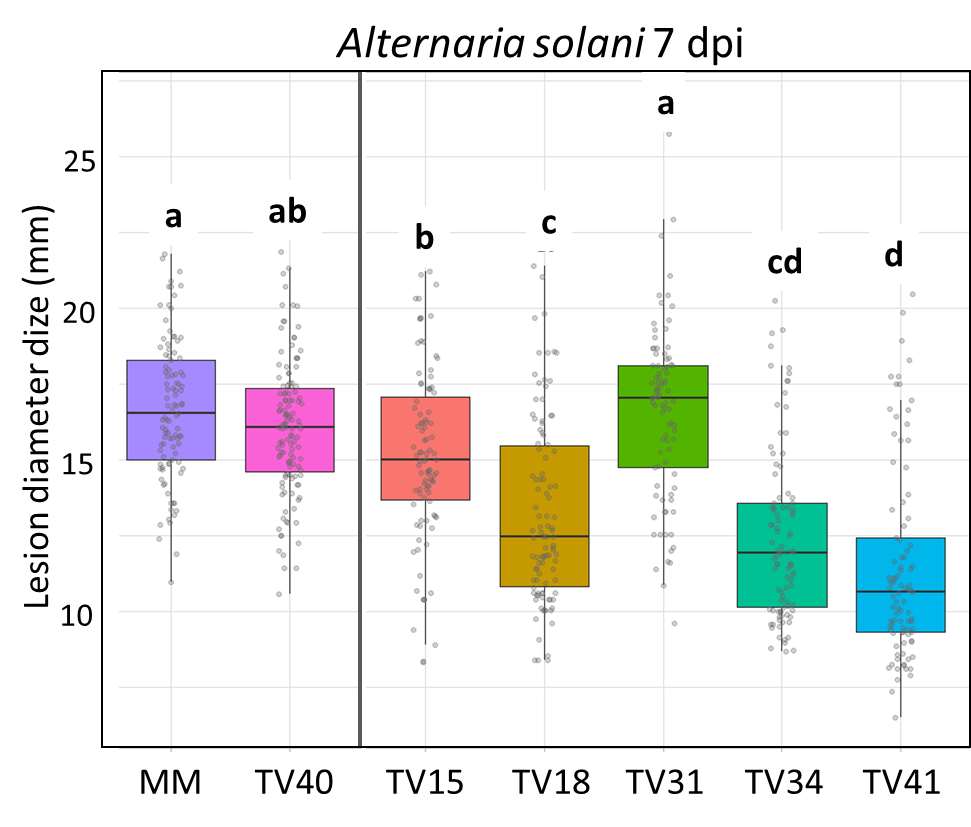
**

**Fig. S4**. Boxplot of *Alternaria solani* lesion diameters on leaves from *PUB21* RNAi T3 families with the two negative controls (TV202240 and MM) on the left of each panel, results from 7 dpi.


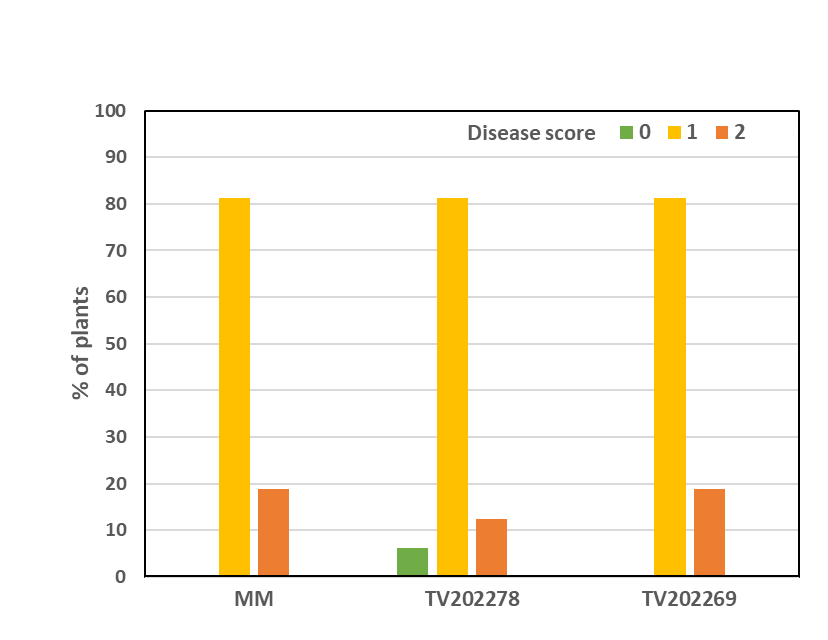


**Fig. S5** Powdery mildew disease scoring of *PUB21* CRISPR mutants. Plants from homozygous mutant TV202269, with susceptible controls Moneymaker and non-mutant T3 family TV202278 were inoculated with a spore solution of *Pseudoidium neolycopersici* isolate On. Disease was scored on a scale from 0 (no symptoms) to 2 (susceptible).

**Supplementary table**

**Table S1**. List of primers

| **Primer Name** | **Primer Sequence 5’→3’** | **PCR product size** | **Used for** |
| --- | --- | --- | --- |
| SP_F | TGAGACGGACAAGATGACATGA | 218 bp | Self-pruning (*sp*) SNP |
| SP_R | TGTCATTTCCCCTTCCAAAGT |  |  |
| C | GGAACTTGGTGTAGCAGAAATTTCCACATTTC | 243 bp | Dwarf (*d*) SNP Marti et al., 2006 |
| D | TTAGTGAGCTGAAACTCTAATCCGTAGAC |  |  |
| AWPUB21F | CATCAAGTGAAAATAACAAGAA | 248 bp | Confirmation *PUB21* SNP |
| AWPUB21R | CAAAATTGAAGTTGAACATTC |  |  |
| PUB17_qPCR_Fw1 | GGAAGTGAAGGTGTTGCGA | 100 bp | *PUB17* gene expression |
| PUB17_qPCR_Rv1 | CTACTGCCATTTCCTCATTGC |  |  |
| PUB21_qPCR_Fw | TGAAGAAGGGAAACAAAAGGCT | 100 bp | *PUB21* gene expression |
| PUB21_qPCR_Rv | AGTTGAACATTCTGTGGCCA |  |  |
| Ef1a-Fw | ATTGGAAACGGATATGCCCCT | 101 bp | Reference gene expression |
| Ef1a-Rv | TCCTTACCTGAACGCCTGTCA |  |  |
| PUB21 RNAi1 FWD | caccATTGAAGCTCGACGAGGGAA | 195 bp | *PUB21* RNAi fragment 1 |
| PUB21 RNAi1 REV | CGTCATCGCCGATAACAAGT |  |  |
| PUB21 RNAi10 FWD | caccCGGTGATATACTCTATTATCTC | 205 bp | *PUB21* RNAi fragment 10 |
| PUB21 RNAi10 REV | GTCAATCCATGTTCATAAGC |  |  |
| PUB21 sgRNA1 | AAACATCGAGAAATGGATCG |  | *PUB21* CRISPR guides |
| PUB21 sgRNA2 | AATCGATTCGTCTCAAGTAA |  |  |
| PUB21 sgRNA3 | GATAGAGTGGATTGCTTTGA |  |  |
|  | TGTGGTCTCA[sgRNAsequence]GTTTTAGAGCTAGAAATAGCAAG |  | Cloning CRISPR guides |
|  | TGTGGTCTCAAGCGTAATGCCAACTTTGTAC |  |  |
| FWD_MR_GY_CRISPR | TCCATCTCATTTTCTTTGTCCGA | 1358 bp | Identification CRISPR mutants |
| REV_AW_GY_CRISPR | TGCTGAGATCCTCCAAAACTATCA |  |  |
| AWPUB21F2 | AATAAATTCACTTTTCCCATATA | 705 bp | Identification CRISPR mutants |
| AWPUB21R2 | GCCGATAACAAGTCCTTC |  |  |
| NPTII_421_Fw | GAAGGGACTGGCTGCTATTG | 421 bp | Confirmation transformants |
| NPTII_421_Rv | AATATCACGGGTAGCCAACG |  |  |
| 35S_597_Fw | TACAAAGGCGGCAACAAAC | 597 bp | Confirmation transformants |
| 35S_597_Rv | AGCAAGCCTTGAATCGTCC |  |  |
